# Acute Hepatitis E: Two Sides of the Same Coin

**DOI:** 10.3390/v8110299

**Published:** 2016-11-03

**Authors:** Johannes Hartl, Malte H. Wehmeyer, Sven Pischke

**Affiliations:** First Medical Department, University Medical Center Hamburg-Eppendorf, University Hospital Hamburg Eppendorf (UKE), 20246 Hamburg, Germany; j.hartl@uke.de (J.H.); m.wehmeyer@uke.de (M.H.W.)

**Keywords:** hepatitis E, HEV, ACLF, liver failure, decompensation

## Abstract

The relevance of acute hepatitis E virus (HEV) infections has been underestimated for a long time. In the past, HEV infection had been interpreted falsely as a disease limited to the tropics until the relevance of autochthonous HEV infections in the Western world became overt. Due to increased awareness, the incidence of diagnosed autochthonous HEV infections (predominantly genotype 3) in industrialized countries has risen within the last decade. The main source of infections in industrialized countries seems to be infected swine meat, while infections with the tropical HEV genotypes 1 and 2 usually are mainly transmitted fecal-orally by contaminated drinking water. In the vast majority of healthy individuals, acute HEV infection is either clinically silent or takes a benign self-limited course. In patients who develop a symptomatic HEV infection, a short prodromal phase with unspecific symptoms is followed by liver specific symptoms like jaundice, itching, uncoloured stool and darkened urine. Importantly, tropical HEV infections may lead to acute liver failure, especially in pregnant women, while autochthonous HEV infections may lead to acute-on-chronic liver failure in patients with underlying liver diseases. Immunosuppressed individuals, such as transplant recipients or human immunodeficiency virus (HIV)-infected patients, are at risk for developing chronic hepatitis E, which may lead to liver fibrosis and cirrhosis in the long term. Importantly, specific treatment options for hepatitis E are not approved by the regulation authorities, but off-label ribavirin treatment seems to be effective in the treatment of chronic HEV-infection and may reduce the disease severity in patients suffering from acute liver failure.

## 1. Introduction

Hepatitis E virus (HEV) infection is a worldwide distributed cause of viral hepatitis. HEV is hyperendemic in many tropical nations (Table 1), and is responsible for numerous outbreaks of hepatitis in these countries, particularly after major floods or in refugee camps [1,2,3]. It is estimated that the number of symptomatic HEV infections in the tropics exceeds 3 million annually, causing approximately 70,000 deaths each year [3].

In Western countries, HEV has been considered a rare, travel-associated and self-limiting liver disease. However, studies published over the last decade demonstrate that this notion was mistaken and autochthonous HEV infections in industrialized countries are far more common than previously suspected [4].

Traditionally, four different human pathogenic HEV-genotypes have been differentiated. However, in 2016, HEV was classified into seven different genotypes based on reference sequences [5].

While HEV infections in the tropics are caused by HEV genotypes (GT) 1 and 2, autochthonous HEV infections in high income countries are mainly caused by HEV GT 3 and 4, which features different clinical characteristics rather than tropical genotypes. For instance, HEV GT 1 and 2 are obligate human pathogens and mainly transmitted via contaminated drinking water in regions with low sanitation, while zoonotic transmission, predominantly by infected swine meat, seems to be the major source of HEV GT 3 and 4 transmission [4,6,7]. In addition, it has been shown that either people in contact with animals or forestry workers have a higher risk for anti-HEV immunoglobulin G (IgG) positivity, demonstrating that these present risk factors for contact with HEV [8]. Moreover, HEV GT 1 infection is associated with fulminant hepatitis and fatal outcomes in pregnant women, but no such association has been observed in zoonotic hepatitis E.

The current review aims to summarize the clinical course of acute hepatitis E in immunocompetent and immunosuppressed individuals, point out the special features of travel-associated and autochthonous HEV infections, and provide implications for HEV-testing and antiviral treatment.

## 2. History of Hepatitis E

Hepatitis E has been well-known since the 1990s, but reports of hepatitis E outbreaks might date back as far as 1794 [9]. Within these outbreaks of hepatitis of unknown-origins, an increased mortality of pregnant women compared to men and non-pregnant women has been observed.

In 1983, the Russian scientist Balayan infected himself orally by ingestion of pooled stool extracts from patients with non-A/non-B hepatitis originating from an Indian hepatitis outbreak in 1955. Thereafter, virus-like particles were identified in his stool samples shortly before and during the clinical phase of hepatitis. This was the first description of HEV-virions. The enteric pathogen could be transmitted to cynomolgus macaques, which induced hepatitis. An RNA species was identified from livers of infected animals and named hepatitis E virus [10].

## 3. The Virus and Its Epidemiology

HEV is a non-enveloped, single-stranded RNA virus (7.2 kb). Initially, the virus has been falsely classified into the group of Caliciviruses, but it is now classified into the family of *Hepeviridae* and its own genus *Hepevirus* [4]. Conventionally, the genome of HEV contains three Open-Reading-Frames (ORF 1–3) [4]. These ORFs contain the genetic information coding for various proteins that are relevant for capsid formation, virus replication and infectivity of HEV [21,22].

Via phylogenetic analysis based on a hypervariable region within ORF1, it is possible to differentiate between various HEV isolates [23]. Previously, four different humanopathogenic HEV-genotypes (HEV GT 1–4) and 24 subtypes (1a–1e, 2a, 2b, 3a–3j, 4a–4g) have been separated [24]. However, based on the identification of HEV-strains from rabbits, wild boars and camels, a novel classification separated seven HEV-genotypes and various subtypes [5]. Surely, this novel classification will gain in importance within the next several years, but, recently, the majority of studies still focus on the four major subtypes (GT 1–4).

In contrast to hepatitis A virus (HAV) genotypes, HEV GT 1–4 show a specific geographical distribution.

HEV genotype 1 is responsible for most endemic and epidemic cases of hepatitis E in Asia and genotype 2 is prevalent in Central America and Africa [4]. HEV GT 1 and 2 are obligate human pathogens and transmission occurs via the oral-fecal route.

HEV GT 3 and 4 have been found in humans and various animals, especially swine, in Europe, the US and Asia [24]. The predominant genotype in Europe is GT 3, especially subtypes 3c, 3e and 3f. Subtype 3a can be found in Asia and the US. For a long time, it has been assumed that GT 4 is limited to Asia, but recent reports also identified this virus in swine and humans in Europe [25].

Zoonotic transmission via infected swine meat has been assumed to be the most relevant source of HEV infection in industrialized countries, as GT 3 and 4 are able to infect humans and swine [4,6,26]. The virus gets inactivated by heating over 70 °C. Therefore, well-cooked swine meat is unsuspicious as an HEV transmitter [27].

A recent report from China discovered that HEV GT 4 is also excreted into milk and demonstrated that HEV-contaminated raw and even pasteurized milk resulted in active infection in rhesus macaques, while short time boiling inactivates the virus [28]. Thus, milk products may be an important source of HEV GT 4 infection in China, but the relevance of milk products as a source of HEV GT 3 infections in Europe remains to be assessed.

**Figure 1:**
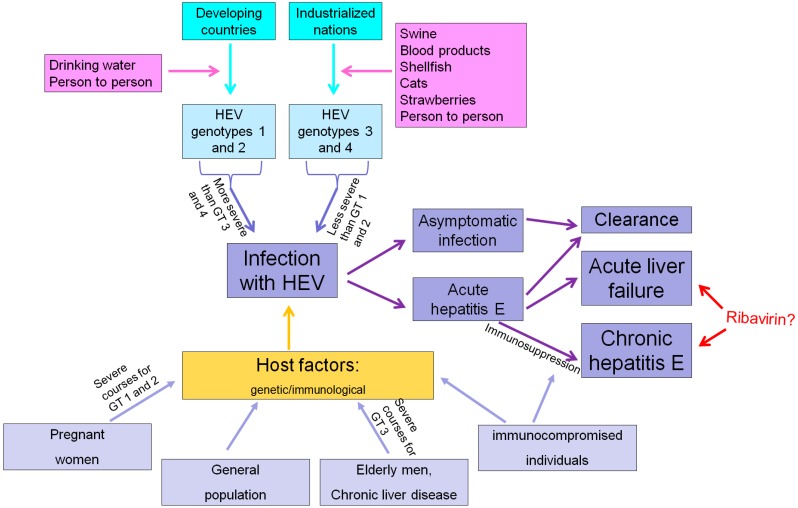
Possible courses of hepatitis E virus (HEV) infection. GT, genotype.

In addition, wild boar, oysters, shellfish, deer, cats, rats, various rodents and camels are also potential hosts for HEV (Figure 1) [4,29,30], but, again, the relevance of these animals as sources of zoonotic HEV transmission is still unclear [31].

Another important route of HEV-transmission in industrialized countries is the transfusion of infected blood products. Within the last few years, several studies have investigated this possible way of transmission [32,33,34,35,36]. In summary, one out of 1000 to one out of 10,000 European blood donors tested positive for HEV-viremia [37]. The most relevant study regarding this topic was published in “*The Lancet*“ in 2014. Hewitt et al. tested 250,000 blood donations from England for the presence of HEV RNA [32]. In total, 79 of them (0.04%) tested positive for HEV RNA, and, in 42% of patients who received an HEV-infected blood product, evidence of hepatitis E could be observed. Furthermore, plasma products are also a possible source of infection, especially in transplant recipients undergoing plasma exchange [38,39]. This aspect is of special relevance as many immunosuppressed individuals, such as kidney or heart transplant recipients, receive plasma products by plasmapheresis. Sometimes, plasmapheresis requires plasma pooled from many donors (e.g., up to 100 in France) [38]. The usual solvents and detergents to inactivate infectious agents are not effective against HEV and thus pooled blood products dramatically increase the risk of HEV transmission [38]. In Europe, HEV-testing of blood-products have still not been generally implemented and this topic is still under debate.

In contrast to zoonotic transmission and transmission via blood products, there are only single reports about HEV transmission directly between humans [40,41], via transplant organs [42] or via strawberries [43]. These possible sources of infection require further investigations to determine their relevance.

## 4. Acute Hepatitis E: An Emerging Disease in Western Countries?

**Figure 2:**
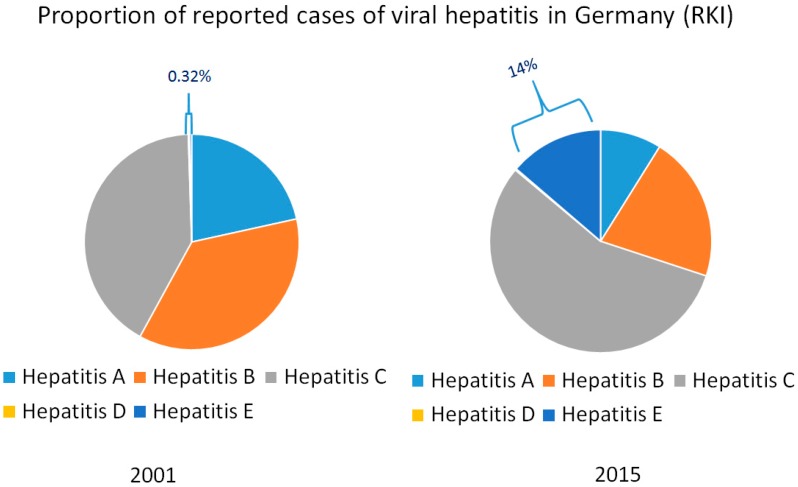
Proportion of diagnosed cases of hepatitis A–E in Germany basing on data from the Robert-Koch-Institute (RKI).

The notably high frequency of HEV RNA in urban sewage samples from Spain, the US, France and Israel clearly highlights an environmental presence of HEV [44,45]. In addition, anti-HEV IgG seroprevalence studies from France and the Netherlands found seroprevalence rates of more than 30% [8]. Nevertheless, anti-HEV-IgG seroprevalence has declined in various studies from different industrialized nations over the last 20 years [46,47,48,49]. This contrasts with the rapidly increasing number of reported HEV infections in several industrialized countries over the last decade. Consequently, HEV infections have become the most frequent cause of acute viral hepatitis in countries such as Germany (Figure 2) and the UK. The discrepancy between decreasing seroprevalence rates and increasing numbers of reported cases indicate that this phenomenon is based on an increased awareness for HEV infections [50]. Based on this assumption, the number of scientific articles on HEV infections has strongly increased since 2006, while i.e., the number of publications on hepatitis A has remained stable [4,47]. Hence, it seems that zoonotic HEV infection in industrialized countries is not emerging but until recently has been an underestimated disease that has, in fact, always been with us.

## 5. Diagnosis of Acute HEV Infection

Early epidemiological studies, which found anti-HEV IgG seroprevalance rates of 1%–2% in Western countries [47], underestimated the disease burden of autochtonous hepatitis E due to limited sensitivity of the anti-HEV-assays [8]. Until now, the question of which assay should be used and who should be tested for HEV infection is controversial [8].

Several anti-HEV-antibody assays for detection of anti-HEV IgG and IgM are available [8]. Importantly, none of these anti-HEV-assays has been approved by the Food and Drug Administration (FDA), while in Europe and Asia, many different assays are used. In the US, testing for HEV is limited to few specialized centers, and thus diagnosis of HEV infections in the US can be delayed [51].

It still needs to be determined, which seroassay has the highest specificity and sensitivity. A large meta-analysis recently published in *Viruses* highlighted that the observed heterogeneity in seroprevalence rates in Europe is mainly attributed to the assay employed [8].

Within the last few years, a novel assay from China (manufacturer: Wantai) has been reported to have a good sensitivity and specificity and is favored by many scientists worldwide [52]. The Wantai assay shows a seroprevalence rate of approximately 20%–30% for anti-HEV-IgG in healthy individuals from high income countries, which is much higher compared to other assays [52,53]. For a long time, it has been questionable if 20%–30% presents a fair or overestimated seroprevalence rate for HEV infections, which only rarely become symptomatic. However, 0.04% of blood donors in England tested positive for HEV-RNA [32]. If we assume a maximum duration of viraemia of six weeks, 0.36% of blood donors in England will be viraemic within one year. Based on this assumption, 18% of the population of England had been HEV-viraemic within the last 50 years. These calculations indicate that a seroprevalence rate of more than 20% in industrialized countries does not overestimate the rate of HEV-exposed persons.

In general, anti-HEV IgG tests indicate previous contact with HEV. Therefore, these antibodies are present in patients after HEV infection and occasionally in patients with an ongoing HEV infection. Anti-HEV IgM assays indicate acute or recent infections and should disappear in patients after exposure to HEV. However, the use of these IgM-assays is still doubtful, as specificity and sensitivity are still unclear.

The gold standard for detection of ongoing HEV infection (acute or chronic) is testing for HEV-RNA by polymerase chain reaction (PCR). In 2011, a World Health Organization (WHO) standard of HEV-RNA has been evaluated, which allows a comparison of qualitative and quantitative PCR assays worldwide and among different laboratories [54].

Testing of stool via PCR might be far more sensitive for the detection of HEV-RNA than testing for viremia (unpublished data by our own group). However, due to practicability and patients’ acceptance, PCR-testing of blood samples is more feasible and used more often.

Within the last few years, a novel assay for the detection of HEV-antigen has been developed [55]. However, the diagnostic fidelity of this assay still needs to be studied prospectively in larger cohorts.

## 6. Clinical Course of HEV Infection

The vast majority of contacts with HEV leads to clinical silent seroconversion and symptomatic hepatitis E develops only in a minority of patients (Figure 1) [17,21,56]. In an HEV vaccine trial in China, which included more than 110,000 individuals, less than 5% of individuals who seroconverted to anti-HEV positivity during the study period developed symptoms of acute hepatitis [16,17]. Furthermore, HEV infections are only rarely symptomatic during childhood [57].

In patients who experience a symptomatic HEV infection the incubation period ranges from three to eight weeks with a mean of 40 days [4]. Peak alanine transaminase (ALT) levels usually can be expected roughly six weeks after infection [4]. There are no typical symptoms that allow differentiation of acute hepatitis E from other forms of viral hepatitis. Similar to hepatitis A, B or C, a short prodromal phase with unspecific symptoms, such as flu-like myalgia, arthralgia, weakness and vomiting, is followed by liver-specific symptoms like jaundice, itching, uncoloured stool and darkened urine. An increase of ALT and aspartate transaminase (AST), accompanied by an increase of alkaline phosphatise (AP), gamma-glutamyl-transferase (γGT) and bilirubin levels is usually found in the routine laboratory. Importantly, ALT levels are typically higher than AST levels.

Individual host factors, which might protect from the development of clinical overt hepatitis are unclear and further research is highly warranted for this important topic (Figure 1). Zhang et al. assessed the association between anti-HEV IgG seropositivity and human genetic variants and found that apolipoprotein ε3 and ε4 variants were associated with a lower HEV seroprevalence rate and might therefore be protective against HEV infection [58]. However, no differences regarding the distribution of these variants between patients with silent seroconversion and patients suffering from clinical overt hepatitis has been observed, and thus this observation should not been interpreted as a protective factor against development of symptomatic hepatitis E [59].

Besides individual host factors, the natural history of HEV infection depends on the viral genotype (Figure 1). In addition, in three groups of patients, the disease course and prognosis are different: immunosuppressed patients are at risk to develop chronic hepatitis, while HEV infection in patients with underlying chronic liver disease and in pregnant women (with HEVGT 1 infection) is associated with a poor prognosis. Thus, the impact of these factors will be discussed in more detail.

## 7. Tropical and Autochthonous HEV Infections May Lead to Different Courses of Acute Hepatitis E

As described above, features of HEV infection in developing and industrialized countries differ regarding the mode of transmission: while autochthonous HEV GT 3 infections in high income countries may result from consumption of contaminated products, tropical HEV-GT 1 or 2 infections are mainly transmitted fecal-orally by contaminated drinking water [4].

**Figure 3:**
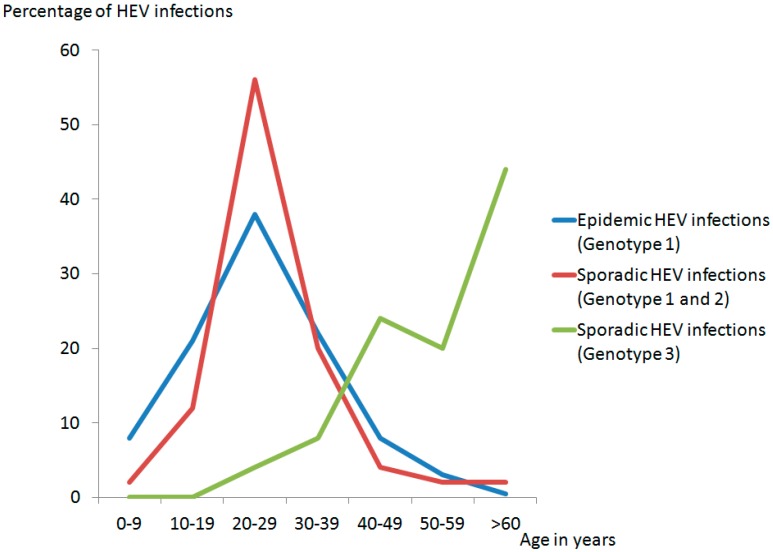
Distribution of HEV infections in different age groups (based on: Purcell and Emerson [61]).

Besides the mode of transmission, HEV-genotypes display several different clinical features. It has been shown that tropical epidemic cases and tropical sporadic cases of hepatitis E (HEV GT 1 and 2) more frequently affect younger people than sporadic HEV GT 3 infections in high-income countries, which affect primarily middle-aged/elderly male (Figure 3) [60].

Additionally, tropical HEV GT 1 and 2 may be associated with a higher manifestation index and a more severe disease course [11]. For example, in a hepatitis E GT 1 outbreak in a German tourist group, five of 24 (20%) individuals developed clinical signs of acute hepatitis E [13], which is in line with an estimated manifestation index of 16% in tropical HEV-infection [56]. In contrast, individuals with HEV GT 3 or 4 infections might develop symptomatic hepatitis E in less than 2% [16,17]. Most recently, we were able to demonstrate that imported HEV infections are associated with increased peak values of ALT, bilirubin, and international normalized ratio (INR), which might provide further evidence that imported tropical HEV GT 1 and 2 infections take more severe courses as compared to autochthonous GT 3 infections in industrialized countries [11].

In recent years, there is increasing evidence for extrahepatic manifestations of HEV infections. A causal pathophysiological link between HEV and these diseases remains to be proven. However, it is most likely that Guillain–Barre syndrome, neuralgic amyotrophy, glomerulonephritis, cryoglobulinemia, and pancreatitis are associated with HEV-infection [61]. Some of these extrahepatic manifestations seem to be restricted to specific HEV-genotypes. For instance, more than 50 cases of HEV-related courses of acute pancreatitis have been reported and all cases occurred in areas endemic for HEV GT 1 [62], while renal manifestations were predominantly reported in HEV GT 3 infections [63]. Extrahepatic manifestations of HEV-infection will be discussed in detail in a separate article of this issue.

An important difference between tropical and autochthonous HEV-infection concerns the disease course in pregnant women and immunosuppressed individuals: on the one hand, HEV GT 1 infections may lead to severe acute liver diseases in pregnant women [64,65,66], which may result in fulminant hepatic failure and death. On the other hand, GT 3 infections in immunosuppressed individuals sometimes lead to the development of chronic hepatitis E, in particular in solid organ transplants [67,68].

There are few studies that have addressed the issue of differences in pathogenicity between HEV GT 3 and 4 infections. In a small cohort of nine French patients with HEV GT 4 infection, a more severe clinical presentation was observed when compared to HEV GT 3 infections [14]. This is in line with a Japanese study, in which patients with HEV GT 4 infection displayed higher ALT levels than those with HEV GT 3 infection [15].

## 8. Acute and Chronic Hepatitis E in Immunosuppressed Individuals

Chronic courses of hepatitis E have been reported in various cohorts of European solid organ transplant recipients since 2008 [67,69,70,71]. Initially, Kamar et al. reported 14 cases of acute hepatitis E in kidney- and liver-transplanted recipients [67]. Eight of these patients showed a progression into chronic hepatitis E with chronically elevated ALT levels, significant histological activity and development of fibrosis within 12 months.

In a multi-center analysis, 66% of solid organ transplant recipients infected with HEV developed a chronic hepatitis E infection [72], but a single-center study on heart transplant recipients found a rate of 21% [73]. Further studies have shown a rate of chronification of approximately 50% in transplant recipients [74,75].

The risk of chronification of HEV infection in transplant recipients may depend on the immunosuppressive drugs taken by the patients. An association between tacrolimus and the risk of developing a chronic HEV infection has been demonstrated [72], and mycophenolate mofetil might protect from chronification [73]. Interestingly, the ability of calcineurin inhibitors to stimulate HEV replication and of mycophenolate mofetil to inhibit HEV replication has been confirmed in vitro [76].

Importantly, in contrast to chronic HEV infection, no cases of HEV-affiliated acute fulminant liver failure have been reported in a transplant recipient.

Furthermore, chronic hepatitis E has been observed in patients infected with the human immunodeficiency virus (HIV) [6,77]. Recently, it has been demonstrated that HEV infection established under the condition of immunosuppression may persist in HIV infected patients despite improvement of their immune status [78].

In addition to transplant recipients and patients with HIV infection, chronic HEV infections have been observed in patients with different underlying conditions of immunosuppression including systemic lupus erythematodes, granulomatosis, retroperitoneal fibrosis or CD4 T cell deficiency [79]. Chronic courses of HEV infection were also found rarely in immunocompetent individuals [80,81,82]. In some of these “assumed immunocompetent individuals” further diagnostic work-up revealed a previously unknown disturbance of the immune system [79].

Recently, it has been demonstrated that the time between HEV infection by blood transfusion and increase of ALT levels in immunosuppressed patients ranges from 50 to 60 days [39].

## 9. Acute HEV-Infection in Pregnant Women

Several studies from developing countries have shown excess mortality in pregnant women. Mortality ranges from 20% to 25% and usually occurs in the third trimester [4]. As described, the excess mortality in pregnancy is restricted to HEV GT 1 and 2 and not seen with GT 3 and 4, nor is it seen with other hepatotropic viruses [83]. The reason for the excess of maternal mortality in HEV infection remains controversial and has been the subject of debate. Possible explanations for the more severe course in pregnant women are hormonal, genetic and immunological changes during pregnancy [12,84,85]. It has been suggested that a reduced expression of the progesterone receptor or a mutation of the human methylenetetrahydrofolate reductase (MTHFR) gene might be associated with development of fulminant hepatitis E in pregnant women [84,85]. The clinical course and pathophysiological aspects of HEV infections during pregnancy are covered in more detail elsewhere in this issue, too.

## 10. Acute HEV Infection in Patients with Underlying Chronic Liver Disease

Chronic liver disease can be caused by several underlying diseases, such as alcohol abuse, chronic hepatitis B or C virus infection, non-alcoholic steatohepatitis, autoimmune liver diseases, or hereditary diseases (e.g., haemochromatosis or Wilson’s disease).

Patients with underlying chronic liver disease, who are infected with hepatitis E, are at risk to develop acute-on-chronic liver failure. In a large Indian cohort of patients with chronic liver disease, hepatic decompensation due to HEV infection was related to a worse outcome as compared to decompensation due to another cause and the 12-month mortality was as high as 70% [86]. Small studies from developed countries also report a poor prognosis in patients with underlying chronic liver disease [7,87,88]. However, the frequency of acute-on-chronic liver failure to due to HEV infection in developing countries is still unclear and warrants further investigation on a larger scale.

Acute-on-chronic liver failure caused by acute deterioration of liver function due to acute hepatitis E is a possibly life-threatening complication. Of note, the relevance of hepatitis E as an underlying cause of decompensation will be described in detail elsewhere in this issue.

## 11. Treatment of Acute Hepatitis E Infection

Thus far, no pharmacological treatment for acute or chronic HEV infection has been approved. However, in the vast majority of cases, HEV takes a self-limited course and no treatment is required (Figure 1).

While several case series provided evidence for the value of ribavirin in the treatment of chronic hepatitis E [75,89,90,91], the knowledge on treatment of acute hepatitis E is still limited. In patients suffering from HEV-associated acute liver failure, ribavirin represents a possible (off-label) treatment option [92]. In single cases of HEV-induced acute or acute-on-chronic liver failure, a fatal course of the disease may have been prevented by ribavirin (Table 2). Ultimately, patients receiving ribavirin also achieved a viral clearance [88,92,93].

In pregnant women, ribavirin is contra-indicated due to a possible feto-toxicity of the substance. However, the high mortality in pregnant women with acute liver failure due to hepatitis E raises the question of whether there is or not a role for the use of ribavirin in pregnant women in highly endemic countries, e.g., India. According to clinicaltrials.gov there is an ongoing study in India, which evaluates the use of ribavirin in patients with HEV-induced acute liver failure (NCT01698723). However, pregnant women are excluded from this study.

In conclusion, to date, there are no data available from controlled-randomized trials on the efficacy of ribavirin in patients with HEV-associated acute liver failure. Therefore, patients suffering from liver failure due to acute HEV infection should only be treated at a hepatology center and the initiation of a ribavirin therapy must be decided on a case-by-case basis.

## 12. The Hepatitis E Vaccine: A Role for Prevention of Acute Hepatitis E?

Initially, in 2007, promising data from a large vaccine trial were published in the New England Journal of Medicine [100], but the further development of this vaccine has been stopped. In 2010, another group presented a study on a novel vaccine, which was safe and efficient [16]. This vaccine has been approved for the use in China in 2012, but it is still not available in the US or Europe. Importantly, it is still unclear if this vaccine prevents infections with HEV GT 3. Therefore, the value of this vaccine for its use in industrialized nations has yet to be determined.

## 13. Conclusions

In conclusion, the burden of acute hepatitis E virus infections in the Western hemisphere has been underestimated for a long time. However, due to increased awareness, the incidence of autochthonous HEV infections (predominantly GT 3) has sharply risen in the last decade. The main source of infections in industrialized countries seems to be infected swine meat, while infections with the tropical GT 1 and 2 usually are transmitted fecal-orally by contaminated drinking water. Otherwise, healthy individuals an acute HEV infection is either clinically silent or takes a benign self-limited course with unspecific symptoms in the vast majority of patients. However, patients with underlying liver diseases are at risk to develop acute-on-chronic liver failure. Of note, chronic HEV infection in immunosuppressed individuals seems to be restricted to HEV GT 3 infection, while excess mortality during pregnancy is a unique feature of HEVGT 1 and 2 infection. Moreover, tropical HEV-infection may be distinguished by autochthonous HEVGT 3 infections by various other clinical features, in particular, more severe diseases and a higher manifestation index.

Importantly, specific treatment options for hepatitis E are not approved by the regulation authorities, but off-label ribavirin treatment seems to be effective in the treatment of chronic HEV infection and may reduce the disease severity in patients suffering from acute liver failure. The initiation of treatment should be decided on a case-by-case basis. Therefore, studies regarding the treatment of acute HEV-infections are highly warranted and patients with a fulminant course of the disease should be treated at experienced centers only.

## Figures and Tables

**Table 1:** Differences between hepatitis E virus (HEV) infections in developing and industrialized nations.

	HEV Infections in Developing Countries	HEV Infections in Tropical Countries
Genotypes (GT) (distribution)	GT 1(Asia, parts of Africa) and GT 2 (Mexico, parts of Africa)	GT 3 (USA, South America, Europe, sometimes in Asia) and 4 (Asia, rarely in Europe)
Severity	Imported infections seem to be more severe than autochthonous ones in Europe [11]. More than 16% of HEV GT 1 or 2 infections result in clinical apparent hepatitis E [12,13].	Less severe than imported infections [11]. GT 4 infections eventually less severe than GT 3 infections [14,15]. Less than 2% of HEV infections in industrialized nations lead to clinical apparent hepatitis E [16,17]
Risk groups for acute or acute-on-chronic liver failure	Pregnant women are at risk for development of acute liver failure [12]	Elderly men, patients with underlying liver diseases [18,19]
Risk groups for chronic HEV infections	No chronic hepatitis E caused by GT 1 or 2	GT 3 and 4 can cause chronic hepatitis E in immunosuppressed individuals (transplant recipients, human immunodeficiency virus (HIV)-infected patients, patients with hereditary or acquired immunological disturbances, haematological patients)
Endemicity	Endemic: Outbreaks during conditions of decreased hygienic conditions [3,20]	Sporadic: rare symptomatic cases, but diagnosed with emerging frequency within the last years

**Table 2:** Treatment experiences in patients with acute hepatitis E (chronic infections are not depicted).

Course of Acute HEV-Infection	Treatment and Outcome	References
A patient with beginning acute on chronic liver failure due to HEV GT 3 infection	Successful treatment with 1200 mg ribavirin for 21 days	Gerolami et al. [92]
Two patients with acute HEV GT 3 infections	Two patients (one after kidney transplantation) with acute hepatitis E were treated with ribavirin (200–1000 mg) for 10 days or 3 months	Peron et al. [88]
21 patients with either HEV GT 3 infections and risk factors for liver failure	21 patients were treated with ribavirin 600–800 mg for up to 3 months. All cleared the infection.	Peron et al. [94]
A case of severe HEV GT 1 infection	A patient from Erythrea with fulminant HEV GT 1 infection was successfully treated with ribavirin	Pischke et al. [75]
Acute HEV GT 1 infections in four patients	Ribavirin treatment (200–600 mg/day for 3–24 weeks, median 12 weeks) led to clearance of HEV-infection	Goyal et al. [93]
Myositis in an immunocompetent man with acute hepatitis E	Successful ribavirin treatment (400 mg each 72 h for 3 months)	Mengel et al. [95]
Myositis and Guillain-Barre in a liver transplant recipient with acute hepatitis E	3-month ribavirin treatment by reduction of immunosuppression, intravenous immoglobulins (1 g/kg/day for 2 days) and ribavirin therapy (400 mg/day adapted to his glomerular filtration rate of 40 mL/min). Neurological symptoms improved under therapy.	Del Bello et al. [96]
15 patients with neurological symptoms during acute hepatitis E	2 patients were treated with ribavirin and intravenous immunoglobulin, 1 was treated with ribavirin only, 1 received corticosteroids, 3 were treated with immunoglobulins only and 8 were not treated. In 6 patients (40%), neurological symptoms lasted at last follow-up (range 4–126 weeks): 1 treated with ribavirin and immunoglobulins, 2 with immunoglobulins only and 3 were not treated.	Perrin et al. [97]
Membranous nephropathy in a kidney transplant recipient	Ribavirin treatment for 3 months. Sustained viral response rapidly followed by complete remission of the nephrotic syndrome.	Taton et al. [98]
Thrombocytopenia and membranous glomerulonephritis in an Indian patient	Steroid treatment (prednisolone 80 mg/day) stopped bleeding and improved kidney function.	Ali et al. [99]
